# Identifying the Core Components of Emotional Intelligence: Evidence from Amplitude of Low-Frequency Fluctuations during Resting State

**DOI:** 10.1371/journal.pone.0111435

**Published:** 2014-10-30

**Authors:** Weigang Pan, Ting Wang, Xiangpeng Wang, Glenn Hitchman, Lijun Wang, Antao Chen

**Affiliations:** Key Laboratory of Cognition and Personality (Ministry of Education), Faculty of Psychology, Southwest University, Chongqing, China; Institute of Psychology, Chinese Academy of Sciences, China

## Abstract

Emotional intelligence (EI) is a multi-faceted construct consisting of our ability to perceive, monitor, regulate and use emotions. Despite much attention being paid to the neural substrates of EI, little is known of the spontaneous brain activity associated with EI during resting state. We used resting-state fMRI to investigate the association between the amplitude of low-frequency fluctuations (ALFFs) and EI in a large sample of young, healthy adults. We found that EI was significantly associated with ALFFs in key nodes of two networks: the social emotional processing network (the fusiform gyrus, right superior orbital frontal gyrus, left inferior frontal gyrus and left inferior parietal lobule) and the cognitive control network (the bilateral pre-SMA, cerebellum and right precuneus). These findings suggest that the neural correlates of EI involve several brain regions in two crucial networks, which reflect the core components of EI: emotion perception and emotional control.

## Introduction

Emotional intelligence (EI) is the capacity to process emotional information accurately and effectively, including the ability to monitor one's own and others' feelings and emotions, discriminate among them and use this information to guide one's thinking and actions [1]. There is an increasing body of evidence indicating that EI plays a critical role in daily life. Research has shown that EI can predict successful social interactions [2], job performance [3], mental health [4] and emotional well-being [5]. In contrast, impaired or deficient EI has been linked to certain symptoms, such as substance abuse disorder [6], anxiety and depression [7], [8].

EI is generally considered as a multidimensional construct [1], [9], [10]. Most conceptualizations of this construct address one or more of the following basic components: (i) the ability to be aware of and express emotions; (ii) the ability to be aware of others' feelings; (iii) the ability to manage and regulate emotions; (iv) the ability to realistically and flexibly cope with the immediate situation; and (v) the ability to generate positive affect in order to be sufficiently self-motivated to achieve personal goals [11]. In short, EI includes the ability to engage in sophisticated information processing about one's own and others' emotions and the ability to use this information as a guide to thinking and behavior [12]. Accordingly, the emotional processing and executive control may be two core processes associated with EI.

Previous neuroimaging studies suggested that the various aspects of EI were supported by separate neural substrates. The social cognition network (SCN) facilitates the understanding of others' feelings, thoughts or desires [13]–[15]. The SCN includes the medial prefrontal cortex (mPFC) and the superior temporal sulcus (STS), which show altered activity during face recognition and mental state attribution [14], and the temporoparietal junction (TPJ), which is associated with the process of inferring temporary states such as the goals, intentions, and desires of other people [15]. In addition, the inferior frontal gyrus (IFG), amygdala, anterior cingulate cortex, and anterior insula are also important portions of the SCN [14]. From the perspective of large-scale networks, the salience network (with key nodes of anterior insula and anterior cingulate cortex) and central executive network (with key nodes of dorsolateral prefrontal cortex and posterior parietal cortex) were considered to be two key networks in cognition [16]. Another critical network related to EI is the emotion processing network. Leppänen and Nelson suggested that the neural systems that are involved in processing emotional signals from faces include the amygdala, orbitofrontal cortex (OFC), fusiform gyrus and posterior STS [17]. In addition, the anterior insula and anterior cingulate cortex in the salience network were activated in emotional processing [18]. A recent review revealed that the process of experience and perception of emotion involved in broadly distributed functional networks, such as the salience network, executive network and default mode work [19], [20]. Structural or anatomical imaging studies (e.g., voxel-based morphometry and diffusion tensor imaging) have found more direct relations between EI and regions in the social emotional processing networks, such as the ventromedial prefrontal cortex, STS, insula and fusiform gyrus [21]–[23]. Additionally, the top-down control network involving fronto-parietal and cingulo-opercular control networks [24]–[27] have been linked to control of emotional expression. Specially, the fronto-parietal component seems to initiate and adjust control, while the cingulo-opercular component provides stable set-maintenance, and both are connected to the cerebellar error-network [25], [27]. A psychophysiological interactions analysis indicated that volitional regulation of emotions produced distributed alterations in connectivity between visual, attention control, and default networks [28]. Several voxel-based morphometry studies found that some regions in the top-down control network were linked to EI, such as the frontal and inferior parietal areas, precuneus and cerebellum [23], [29]. A resting state functional connectivity (RSFC) study found that total trait EI was positively correlated with RSFC between the mPFC and the precuneus, as well as between the left anterior insula and the middle part of the right dorsolateral prefrontal cortex [30].

Although previous studies mainly focused on structural or anatomical neuroimaging to investigate the EI-related brain regions and networks, it is necessary to pay more attention to explore the underlying spontaneous brain activity related to EI. The spontaneous fluctuations in the blood oxygen level dependent (BOLD) signal reveal the intrinsic functional architecture of the brain and relate to extrinsic task performance [31], [32]. Moreover, the absence of demanding cognitive activities and instructions makes it more straightforward to compare brain activity across groups that may differ in behavioral performances [33]. Therefore, the task-free resting state spontaneous activity has a unique advantage to investigate the underlying neural basis of EI. The amplitude of spontaneous low-frequency fluctuations (ALFFs) are widely used for measurement of the spontaneous fluctuations in brain activity [34]-[36]. The ALFF has been suggested to reflect the intensity of regional spontaneous brain activity [34], [37]. It has been proved to correlate with task-evoked BOLD responses and is also found to have robust predictive value for behavior [32]. Specially, ALFF has been reported to be associated with cognitive processing abilities (e.g., conceptual processing capacity; object color knowledge performance) [35], [38] and personality traits [39], [40]. Furthermore, ALFF was considered as promising potential biomarkers of mental disease or disorders, such as bipolar disorder [41], amnestic mild cognitive impairment [42], PTSD [33] and Parkinson's disease [43]. These findings suggest that ALFF may be an effective indicator to reflect EI-related spontaneous regional brain activity.

To our knowledge, no study has yet investigated the relationship between the resting state fMRI indicator of ALFF and EI. Thus, in the present study, we explored the ALFFs of resting state fMRI signals to elucidate the intrinsic neural basis of EI. As mentioned previously, EI is a multidimensional construct and includes the ability to understand one's own and others' emotions and the ability to regulate and manage emotions. These different processes may involve in emotional information processing and advanced executive control function of the brain. Therefore, we hypothesized that EI might be linked to several brain regions or networks, such as emotion processing networks, cognitive control networks, salience networks or default mode networks.

## Methods

### Ethics statements

The procedure of this study was approved by the Ethics Committee of the Southwest University. Written informed consent was obtained from all participants. They were informed that the experiment was completely voluntary and they can quit at any time during the experiment. Two of the participants are less than 18 years old, so written informed consent was obtained from their parents on behalf of them.

### Participants

One hundred and seventy participants were recruited to take part in this study. Seven of them exhibited excessive head motion and two failed to register to the standard Montreal Neurological Institute (MNI) space in data preprocessing and thus were excluded. Finally, one hundred and sixty-one individuals were included in the formal analysis. All the participants were healthy, right-handed college students (91 females and 70 males; 19.40±1.28 years old, range: 17–25 years old) with no history of neurological or psychiatric disorders. Each participant was required to complete the emotional intelligence scale immediately after the rest-state scanning.

### Emotional intelligence scale

All the participants completed the Chinese version of the Wong and Law Emotional Intelligence Scale (WLEIS), a 16-item self-report questionnaire designed to measure trait EI [44]. The scale has been demonstrated to be reliable and valid to assess Chinese trait EI [10], [45]–[47]. The WLEIS is comprised of four subscales: (a) self-emotion appraisal (SEA), (b) others' emotion appraisal (OEA), (c) regulation of emotion (ROE), and (d) use of emotion (UOE) [44]. Examples of WLEIS items are as follows: “I have good understanding of my own emotions” (SEA); “I always know my friends' emotions from their behavior.” (OEA); “I have good control of my own emotions.” (ROE); “I always encourage myself to try my best.” (UOE) [44]. All the responses were made on 7-point Likert-type scales (from 1: strongly disagree, to 7: strongly agree). In the present study, the internal consistency reliabilities (Cronbach *α*s) of the total scale and four subscales were 0.92, 0.84, 0.92, 0.70 and 0.90, respectively.

### Image acquisition

MRI data were obtained using a 3.0 Tesla Siemens Trio scanner (Siemens Medical, Erlangen, Germany) in Southwest University, China. First, high-resolution anatomical images were acquired sagittally with the following parameters: 128 slices, 2530/3.39 ms (TR/TE), 1.33 mm (thickness), 256*256 mm (FOV), 1100 ms (inversion time), 7° (flip angle). In addition, an echo-planar imaging sequence was used to collect resting state functional images, and the acquisition parameters were: 33 axial slices; slice thickness, 3 mm; repetition time (TR), 2 s; echo time (TE), 30 ms; image matrix, 64*64; flip angle, 90°; field of view (FOV), 200*200 mm; and 240 volumes. During the resting state scanning, participants were instructed to lay still with eyes closed, and not to think of anything in particular.

### Data preprocessing

The anatomical and functional image preprocessing was performed using SPM8 (Wellcome Department of Conitive Nurology, London, UK, SPM8; http://www.fil.ion.ucl.ac.uk/spm) and Data Processing Assistant for Resting-State fMRI (DPARSF) [48]. The first 10 volumes of the functional images were discarded to ensure the signals approached a dynamic equilibrium. Then, slice timing was used to correct slice order, and head motion correction was performed to estimate and modify the head movements. Seven participants exhibited head motion >2 mm maximum translation and/or 2° rotation throughout the course of scans, so they did not go into the formal data analysis. Then, each participant's anatomical image was coregistered to the mean functional image and was subsequently segmented. Next, the segmented data was used to normalize all the functional images into standard MNI space in 3*3*3 mm voxel sizes. The normalized images were spatially smoothed with an 8-mm full-width at half maximum (FWHM). After the linear trends were removed, the images were temporally band-pass filtered (0.01–0.08 Hz) to reduce low-frequency drift and high-frequency noise [49].

### ALFF analysis

ALFF analysis [34] was performed using the Resting-State fMRI Data Analysis Toolkit (REST 1.8) [50]. According to Zang et al. [34], the time series was transformed to a frequency domain with a fast Fourier transform (FFT) and the power spectrum was then obtained. The obtained power spectrum was square-rooted and averaged across 0.01–0.08 Hz at each voxel, and this averaged square root was taken as the ALFF. The ALFF value of each voxel in the brain was extracted as the sum of amplitudes within the low-frequency range (0.01–0.08 Hz) [35], [38]. To reduce the global volume effects of variability across the participants, the ALFF maps were divided by whole brain mean ALFF values. Because low-frequency fluctuations are sensitive to signals in the gray matter [38], we calculated ALFFs only in the gray mask. Following the methods of Wang et al. [35] and Wei et al. [38], we included voxels with a probability higher than 0.4 in the SPM8 template onto the gray matter mask, and finally there were 53,464 voxels (1,443,528 mm^3^) in the gray matter mask.

### ALFF-EI correlation analysis

A multiple regression analysis was conducted to examine the correlations between the mean ALFF values and the EI scores. We included gender and age as nuisance covariates in this analysis. Because there is no agreed conclusion about the gender effect on EI [51], [52], we also performed the analysis without regressing out gender. We found these results were similar (for additional details about the effect of gender on EI, see Text S1 in File S1). We performed further analysis to examine the moderator role of gender on the relationship between ALFF and EI and found the moderating effect of gender was not significant. To control for Type I errors, AlphaSim was used for multiple comparisons correction. A threshold of corrected cluster *p*<0.05 (single voxel *p*<0.01, cluster size ≥1,647 mm^3^) was set.

## Results

### Behavioral data

The means and standard deviations of WLEIS total score and scores on its four subscales are presented in Table 1. Pearson correlation analysis showed that the correlations among the four subscales ranged from 0.432 to 0.641 (*p*s <0.001) and the correlations between each subscale and the total score ranged from 0.765 to 0.852 (*p*s <0.001).

**Table 1:** Means and SDs of WLEIS total and subscale scores.

	EI-total	SEA	OEA	ROE	UOE
Mean	83.20	21.53	21.08	19.73	20.86
SD	14.73	4.53	4.73	5.04	3.80

### EI-related brain regions

**Figure 1:**
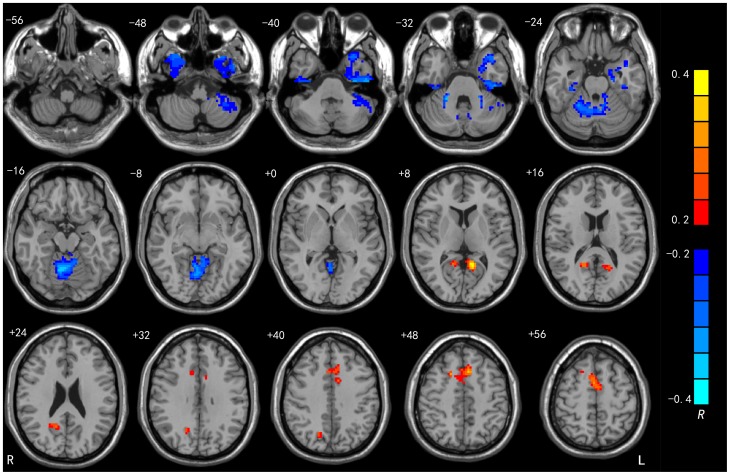
Brain regions which exhibited significant correlations between ALFFs and WLEIS total scores. Color bars represent R values. The results are shown with *p*<0.05 (corrected).

**Figure 2:**
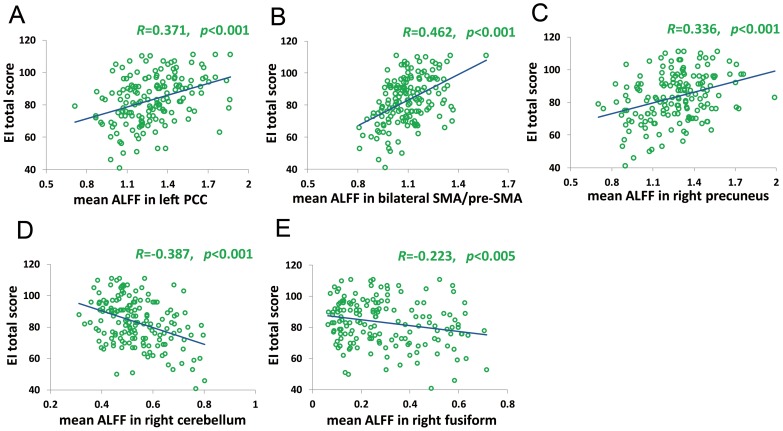
Scatter plots of the relationships between WLEIS total score and mean ALFF values in the significant clusters. A, B, C, D and E showed significant correlations between EI total score and mean ALFFs in left PCC, bilateral SMA/pre-SMA, right precuneus, right cerebellum and right fusiform gyrus, respectively.

We performed regression analysis to explore the correlations between the brain's regional spontaneous activity and EI. As shown in Figure 1, Figure 2 and Table 2, EI total scores were positively correlated with ALFFs in the left PCC (r_peak_  = 0.38, r_cluster_  = 0.37, *p*s <0.001), bilateral SMA (mainly the pre-SMA; r_peak_  = 0.37, r_cluster_  = 0.46, *p*s <0.001) and right precuneus (r_peak_  = 0.29, r_cluster_  = 0.34, *p*s <0.001), and negatively correlated with ALFFs in the right cerebellum (r_peak_  =  −0.41, r_cluster_  =  −0.39, *p*s <0.001) and right fusiform gyrus (r_peak_ =  −0.29, *p*<0.001; r_cluster_  =  −0.22, *p*<0.005).

**Table 2:** Regions in which ALFFs were significantly related with WLEIS in the whole-brain analysis.

Brain regions	BA	Peak MNI coordinates			Peak R	No. of voxels
		x	y	z		
**EI-total**						
L PCC	29	−9	−51	6	0.38	74
B SMA/pre-SMA	6/8	−6	21	51	0.37	272
R precuneus	31/7	18	−54	18	0.29	96
R cerebellum		3	−60	−3	−0.41	1601
R fusiform/temporal pole	37/38	36	15	−48	−0.29	192
**SEA**						
L PCC	29	−9	−51	6	0.37	61
R precuneus	31/7	15	−57	27	0.32	135
B SMA/pre-SMA	6/8	6	9	54	0.28	91
L cerebellum		−15	−36	−30	−0.39	743
L fusiform	37	−39	−24	−30	−0.37	558
R temporal pole	38	33	18	−48	−0.27	156
R fusiform	37	42	−27	−33	−0.27	113
**OEA**						
L inferior parietal lobule	40	−54	−39	54	0.39	80
L inferior frontal gyrus	44/45	−36	39	12	0.33	61
R cerebellum		33	−72	−24	−0.35	551
L cerebellum		−51	−69	−33	−0.31	271
L fusiform	37	−30	−18	−39	−0.26	121
**ROE**						
B SMA/pre-SMA	6/8	−6	21	51	0.42	245
L supramarginal gyrus	40	−54	−24	18	0.36	63
R cerebellum		9	−57	−18	−0.34	285
R superior orbital frontal gyrus	11	12	45	−27	−0.32	85
**UOE**						
B SMA/pre-SMA	6/8	−9	18	51	0.30	122
R precuneus	31/7	21	−51	18	0.29	83
R cerebellum		9	−57	−18	−0.39	679
L temporal pole	38/20	−36	15	−30	−0.36	622
R temporal pole	38/28	39	18	−30	−0.35	77
R fusiform	37	33	−24	−27	−0.29	78

Note: The threshold was set at *p*<0.05 (AlphaSim corrected). BA  =  Brodmann area; B  =  bilateral; R  =  right; L  =  left; SEA  =  self-emotion appraisal; OEA  =  others' emotion appraisal; ROE  =  regulation of emotion; UOE  =  use of emotion. All of the correlation coefficients were significant at the level of *p*<0.001.

Within the subscales of the WLEIS, the following associations were found. As shown in Table 2, ALFFs in the SMA/pre-SMA, cerebellum, fusiform gyrus, right precuneus, left PCC and temporal pole (TP) were significantly associated with most subscales of WLEIS. Whereas, the right superior orbital frontal cortex (OFC) and left supramarginal gyrus (SMG) were merely correlated with ROE and the left inferior parietal lobule (IPL) and left IFG were only correlated with OEA. Specially, the SMA/pre-SMA, precuneus, PCC, SMG, IPL and IFG were positively correlated with EI, but the fusiform gyrus, OFC and TP were negatively correlated with EI. All significant correlations were set at the threshold of corrected cluster *p*<0.05 (single voxel *p*<0.01, cluster size ≥1,647 mm^3^).

## Discussion

In the present study, we performed ALFF-EI correlation analysis to investigate the neural basis of EI. Our results indicated that inter-individual differences in EI were reflected in the ALFFs during resting state. As expected, EI was linked with some regions that are known to be involved in social and emotional information processing to understand emotions of self and others, such as the fusiform gyrus, right superior orbital frontal gyrus, left inferior frontal gyrus and left inferior parietal lobule. Additionally, some regions in the top-down control network, such as the bilateral pre-SMA, cerebellum and right precuneus were associated with EI, which may contribute to the control of emotional expression.

### EI-related brain regions in the social emotional processing network

We found ALFFs in the fusiform gyrus were negatively correlated with EI total, SEA, OEA and UOE scores and ALFFs in the superior OFC were negatively correlated with ROE score. These results indicated that people with high EI showed low spontaneous neural activities in these brain regions. The fusiform gyrus and OFC are core parts of the emotional processing network [17]. The fusiform gyrus is a face-sensitive region [53]. Specially, the activity in this area is enhanced in response to fearful as compared with neutral facial expressions [17], [54]. The OFC is also critical for perceptual processing of emotional signals from faces [17]. Furthermore, OFC has been linked to the experience of anger [55], as well as to aggression [19]. The fusiform gyrus can send visual information to the amygdala and OFC and receive feedback from them [17]. This neural circuit may provide the basis of facial expression cognition and contribute to the understanding and regulation of emotions according to others' feedback in interpersonal communications. It is worth noting that ALFFs in the fusiform gyrus and OFC were negatively related to EI. Given that the fusiform gyrus and OFC, like the amygdala, were associated with negative emotion processing, we suppose that people with low EI would experience more negative emotions. This speculation is in accordance with the finding that impaired or deficient EI was linked to anxiety and depression [7], [8]. These results may contribute to our understanding on the mechanism of susceptibility of depression and social maladjustment of people with low EI. However, further work is required to confirm the mechanism by which the fusiform gyrus, OFC and EI are linked.

In this study, there was a significant positive relationship between OEA score and ALFFs in the IFG and IPL. The IFG and IPL are important parts of the mirror neuron system [56], [57]. There is a “mirror” system in the brain such that the same areas are activated when we observe another person experiencing an emotion as when we experience the same emotion ourselves, so we can experience the emotional states of another person [58]. A lot of research revealed the mirror system's role in social cognition and its contribution to understanding the actions and intentions of other individuals [59], [60]. In line with these findings, our results showed that increased spontaneous brain activity in the IFG and IPL was associated with higher OEA. The IFG and IPL are important nodes of the social cognition network [14], [15], [61]. Posterior regions of the IFG are involved in emotional judgement and might have a role in top-down aspects of emotion recognition [62]. By contrast, the IPL might play a role in high-level mental state inference, such as understanding others' behavior in terms of internal beliefs, feelings, goals, and intentions [15], [61].

Taken together, we provide evidence that EI was significantly associated with ALFFs in certain regions in the social emotional processing network, which is involved in understanding the emotions of self and others.

### EI-related brain regions in the cognitive control network

Our results showed a significant positive correlation between EI total, ROE and UOE scores and ALFFs in the bilateral SMA/pre-SMA. By contrast, EI total and four subscales were negatively correlated to ALFFs in the cerebellum. ALFFs in the right precuneus were positively associated with EI total, SEA and UOE scores. The SMA/pre-SMA, cerebellum and precuneus are key nodes of the cognitive control network [24]–[27]. The SMA/pre-SMA is known to be involved in the processing of task switching [63] and response inhibition [64], [65]. Moreover, the pre-SMA was found to be associated with the control of action [66] and response selection [67]. The pre-SMA was also involved in appraisal and expression of negative emotion [68] and emotional conflict detection [69]. Specially, reappraisal, a cognitive strategy of regulating negative emotions, was reliably associated with activation in the pre-SMA [68], [70]. These findings demonstrate the cognitive and behavioral control functions of the pre-SMA in emotional processing. It is consistent with our finding that increased spontaneous activity in pre-SMA was associated with higher ability of ROE and UOE. We speculate that the activity of the pre-SMA may be conducive to emotional control (to express or inhibit emotion) in complicated interpersonal interactions and further improving the ability of emotional regulation and use.

The cerebellum has neuroanatomical associations with SMA/pre-SMA [71]–[73].The traditional view of cerebellar function is that it is purely involved in motor control and coordination [74]. However, increasing studies suggest that the cerebellum also contributes to cognitive processing and emotional control [72], [73], [75], [76]. A repetitive transcranial magnetic stimulation study found that the inhibition of cerebellar function would lead to increased negative mood as a result of impaired emotion regulation [77]. A meta-analysis suggested that the cerebellum does not play a domain-specific role in social cognition, but most probably provides domain-general executive and semantic support [78]. Besides the social cognition function, the cerebellum is also activated in negative emotion processing, such as anger [79]. Interestingly, the cerebellar activations associated with negative emotions occurred concomitantly with activations of mirror neuron domains, suggesting that the potential role of the cerebellum in control of emotions may be particularly relevant for goal-directed behavior that is required for observing and reacting to another person's negative expressions [80]. In the present study, we found ALFFs in the cerebellum were negatively linked to EI, indicating that low spontaneous activity in cerebellum was related to high EI. This finding is partly consistent with the structural imaging result that the right cerebellum was negatively correlated to the intrapersonal factor of EI [23]. However, it is difficult to interpret why spontaneous activity in cerebellum was negatively related to EI. Because of the multiple functions of brain regions, we suppose that pre-SMA might mainly involve in emotion regulation and control, while the cerebellum tends to be active in negative emotion processing. Future studies should further explore the relationship between the activity in the cerebellum and EI.

The precuneus is in the fronto-parietal control network and is involved in initiating and adjusting control [25], [27]. In our study, the precuneus was positively associated with EI total and UOE scores. This result partly fits with previous evidence that the total EI was positively correlated with RSFC between the mPFC and the precuneus [30]. Moreover, the precuneus was also found to be involved in the processes of emotional regulation [81] and emotional awareness [82]. The ability of emotional awareness and regulation may further improve the efficiency of using emotions. It is worth noting that the SMA/pre-SMA, cerebellum and precuneus are not independent but functionally connected in a larger network [25], [27].

This study may have some limitations. First, the subjects in this study were young college students with high educational backgrounds. Their EI may be higher than the average population. Future studies might use more representative samples to test our findings. Second, although these results provide insight into the neural bases of EI, they only revealed EI-related brain regions, and we do not know whether they work together or independently. Furthermore, a framework that relies on domain general, distributed structure–function mappings emerges recently [83]. Some studies in social cognitive neuroscience revealed that a specific mental activity or trait may involve several domain general networks, such as salience network, central executive network, dorsal attention network and default mode network [20], [83], [84]. Thus, future research may investigate how these networks functionally associate with EI.

In summary, we examined whether individual differences in the amplitude of spontaneous low-frequency fluctuations (ALFFs) during resting state were predictive of variations in EI. We found that several brain regions were significantly associated with EI, including the bilateral fusiform gyrus, right superior OFC, left IFG and left IPL that belong to the social emotional processing network and the bilateral pre-SMA, cerebellum and right precuneus that are in the top-down control network. These regions are involved in understanding and controlling emotions. These findings provide additional evidence of individual differences in brain spontaneous activity linked to EI and deepen our understanding on the core components of EI.

## Supporting Information

File S1 Contains the following files: Text S1. The effect of gender on EI. Table S1. Regions in which ALFF significantly related to WLEIS in the whole-brain analysis. Figure S1. Brain regions exhibited significant correlations between ALFF and WLEIS total score. Figure S2. The common brain regions of Figure 1 and Figure S1.
(DOC)
