# Supplementary material for: Identifying the Core Components of Emotional Intelligence: Evidence from Amplitude of Low-Frequency Fluctuations during Resting State
Source: PLoS One. 2014 Oct 30;9(10):e111435. doi: 10.1371/journal.pone.0111435 (PMC4214743; doi:10.1371/journal.pone.0111435)
Supplement: File S1 — Contains the following files: Text S1. The effect of gender on EI. Table S1. Regions in which ALFF significantly related to WLEIS in the whole-brain analysis. Figure S1. Brain regions exhibited significant correlations between ALFF and WLEIS total score. Figure S2. The common brain regions of Figure 1 and Figure S1. (DOC) [file pone.0111435.s001.doc]

**Supplementary information**

**Text S1 The** **effect of gender on EI**

Gender is an important variable that may affect the relations between ALFF and EI. Some studies [e.g., ] suggested that females had high emotional intelligence than males. But other researchers did not find a gender difference in trait EI [e.g., ]. These results suggest that there is no agreed conclusion about the gender effect on EI.

In order to examine the gender differences on EI, we firstly analyzed the behavioral data. In our study, there were no significant gender differences with respect to the EI total score (t=-0.65, p=0.52) and four subscales (SEA: t=-0.14, p=0.89; OEA: t=-1.78, p=0.08; ROE: t=0.48, p=0.64; UOE: t=-0.79, p=0.43).

We then analyzed the gender effect on EI-ALFF correlation. We examined whether these results were different when gender was regressed out as a nuisance covariate or not. Table S1 and Figure S1 indicated the regions in which ALFF values were significantly related to EI scores when gender was not regressed out. The blue font indicated these results were the same as the results when gender was regressed out. We further analyzed the common regions of Figure 1 and Figure S1 (See Figure S2) and found that they shared most of the brain regions.

These results suggested that gender had little effect on ALFF-EI correlation in the present study.

**Table S1.** Regions in which ALFF significantly related to WLEIS in the whole-brain analysis

| Brain regions | BA | Peak MNI coordinates | | | Peak R | No. of voxels |
| --- | --- | --- | --- | --- | --- | --- |
|  |  | x | y | z |  |  |
| **EI-total** |  |  |  |  |  |  |
| L PCC | 29 | -9 | -51 | 6 | 0.39 | 69 |
| B SMA/pre-SMA | 6/8 | -6 | 21 | 51 | 0.37 | 264 |
| R precuneus | 31/7 | 18 | -54 | 18 | 0.29 | 74 |
| R cerebellum |  | 3 | -60 | -3 | -0.41 | 739 |
| L cerebellum |  | -48 | -15 | -42 | -0.34 | 800 |
| R superior orbital frontal gyrus | 11 | 12 | 45 | -27 | -0.31 | 71 |
| R fusiform | 37 | 42 | -27 | -33 | -0.27 | 177 |
| **SEA** |  |  |  |  |  |  |
| L PCC | 29 | -9 | -51 | 6 | 0.37 | 62 |
| R precuneus | 31/7 | 15 | -57 | 27 | 0.32 | 112 |
| B SMA/pre-SMA | 6/8 | 6 | 9 | 54 | 0.27 | 85 |
| L cerebellum |  | -15 | -36 | -30 | -0.39 | 695 |
| L fusiform | 37 | -39 | -24 | -30 | -0.36 | 522 |
| R temporal pole | 38 | 39 | 15 | -30 | -0.27 | 64 |
| R fusiform | 37 | 42 | -27 | -33 | -0.28 | 120 |
| **OEA** |  |  |  |  |  |  |
| L inferior frontal gyrus | 44/45 | -36 | 39 | 12 | 0.33 | 61 |
| B SMA/pre-SMA | 6/8 | 3 | 21 | 45 | 0.27 | 66 |
| R cerebellum |  | 33 | -72 | -24 | -0.35 | 459 |
| L cerebellum |  | -51 | -69 | -30 | -0.30 | 150 |
| L fusiform | 37 | -30 | -18 | -39 | -0.27 | 172 |
| **ROE** |  |  |  |  |  |  |
| B SMA/pre-SMA | 6/8 | -6 | 21 | 51 | 0.42 | 242 |
| L supramarginal gyrus | 40 | -54 | -24 | 18 | 0.35 | 62 |
| R cerebellum |  | 9 | -57 | -18 | -0.34 | 287 |
| R superior orbital frontal gyrus | 11 | 15 | 45 | -27 | -0.31 | 70 |
| **UOE** |  |  |  |  |  |  |
| B SMA/pre-SMA | 6/8 | -9 | 18 | 51 | 0.30 | 115 |
| R precuneus | 31/7 | 18 | -51 | 15 | 0.30 | 62 |
| R cerebellum |  | 9 | -57 | -18 | -0.40 | 593 |
| L temporal pole | 38/20 | -36 | 15 | -30 | -0.37 | 609 |
| R temporal pole | 38/28 | 39 | 18 | -30 | -0.35 | 103 |
| R fusiform | 37 | 33 | -24 | -27 | -0.31 | 106 |

Age was taken as a nuisance covariate in the ALFF-EI correlation analysis. The threshold was set at p<0.05 (AlphaSim corrected). BA=Brodmann area; B=bilateral; R=right; L=left; SEA=self-emotion appraisal; OEA=others’ emotion appraisal; ROE=regulation of emotion; UOE=use of emotion. All of the correlation coefficients were significant at the level of p<0.001


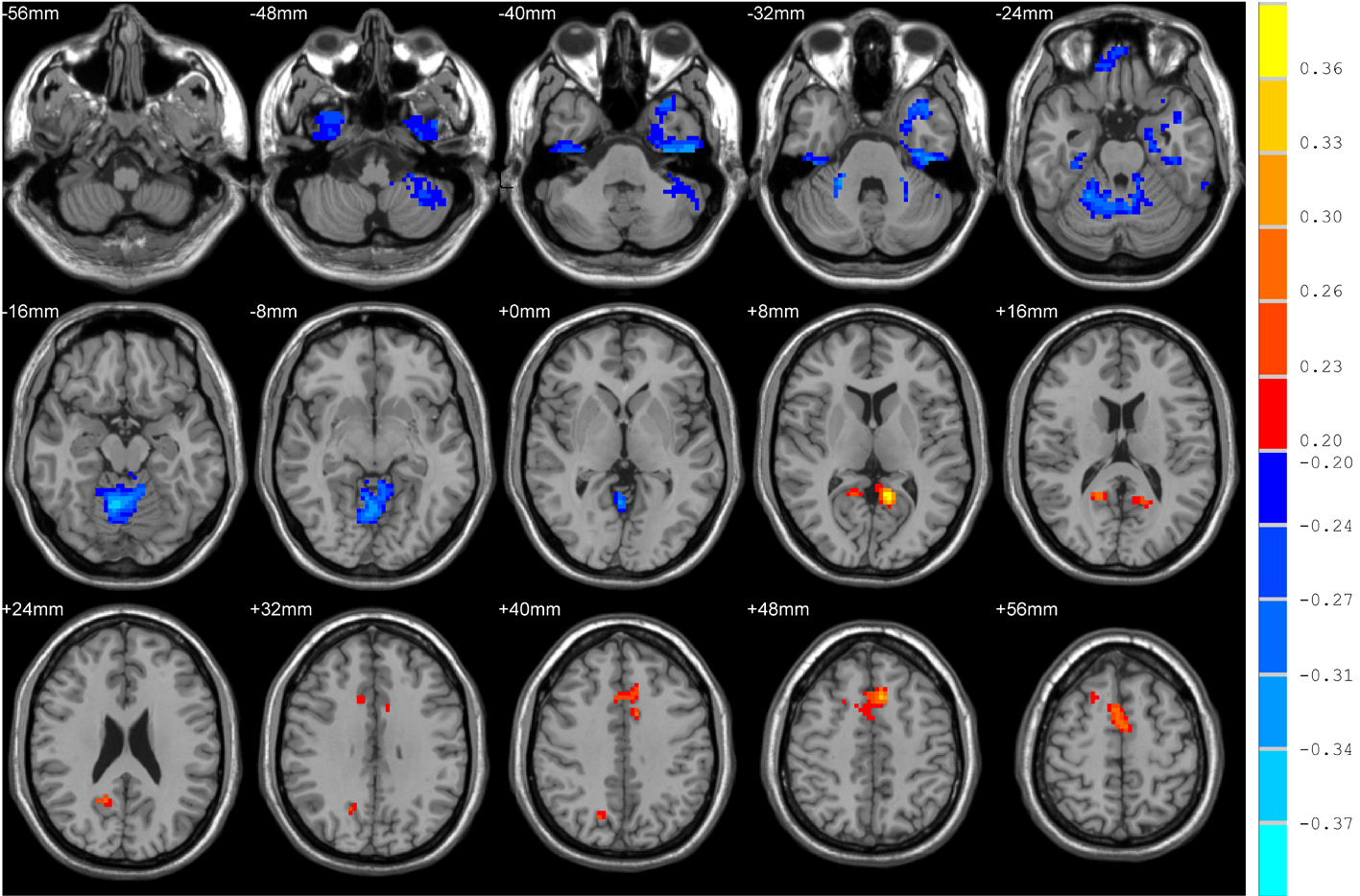


**Figure S1** Brain regions exhibited significant correlations between ALFF and WLEIS total score. Age was taken as a nuisance covariate in the ALFF-EI correlation analysis. Color bars represent R values. The results are shown with p<0.05(corrected).


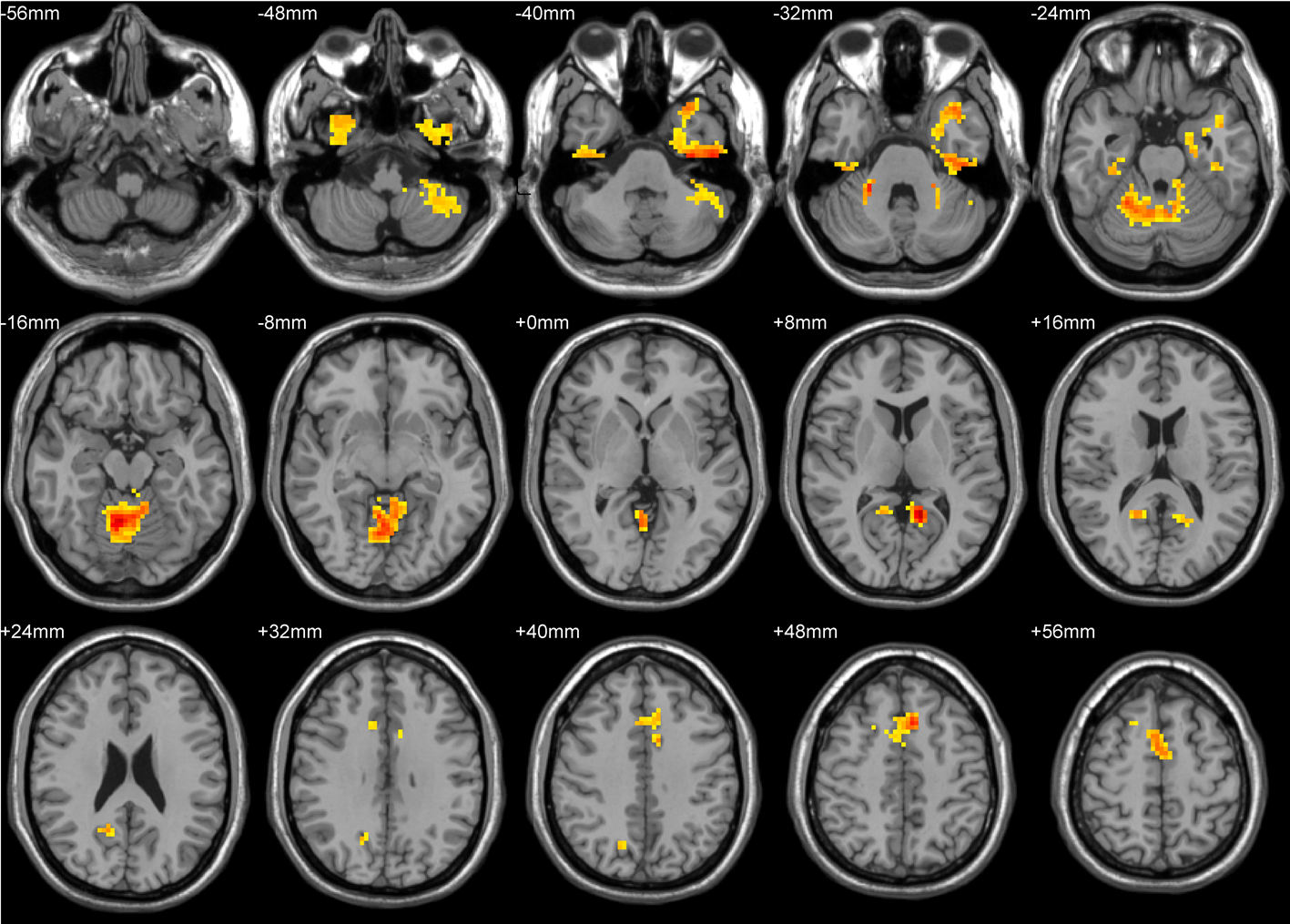


**Figure S2** The common brain regions of Figure 1 and Figure S 1.
